# Supplementary material for: Need for Speed: Examining Protein Behavior during CryoEM Grid Preparation at Different Timescales
Source: Structure. 2020 Nov 3;28(11):1238–1248.e4. doi: 10.1016/j.str.2020.07.018 (PMC7652391; doi:10.1016/j.str.2020.07.018)
Supplement: Document S1. Figures S1–S7 and Tables S1–S4 [file mmc1.pdf]

Structure, Volume *28*

Supplemental Information

Need for Speed: Examining Protein Behavior during CryoEM Grid Preparation at Different Timescales

David P. Klebl, Molly S.C. Gravett, Dimitrios Kontziampasis, David J. Wright, Robin S. Bon, Diana C.F. Monteiro, Martin Trebbin, Frank Sobott, Howard D. White, Michele C. Darrow, Rebecca F. Thompson, and Stephen P. Muench

Supplemental Material

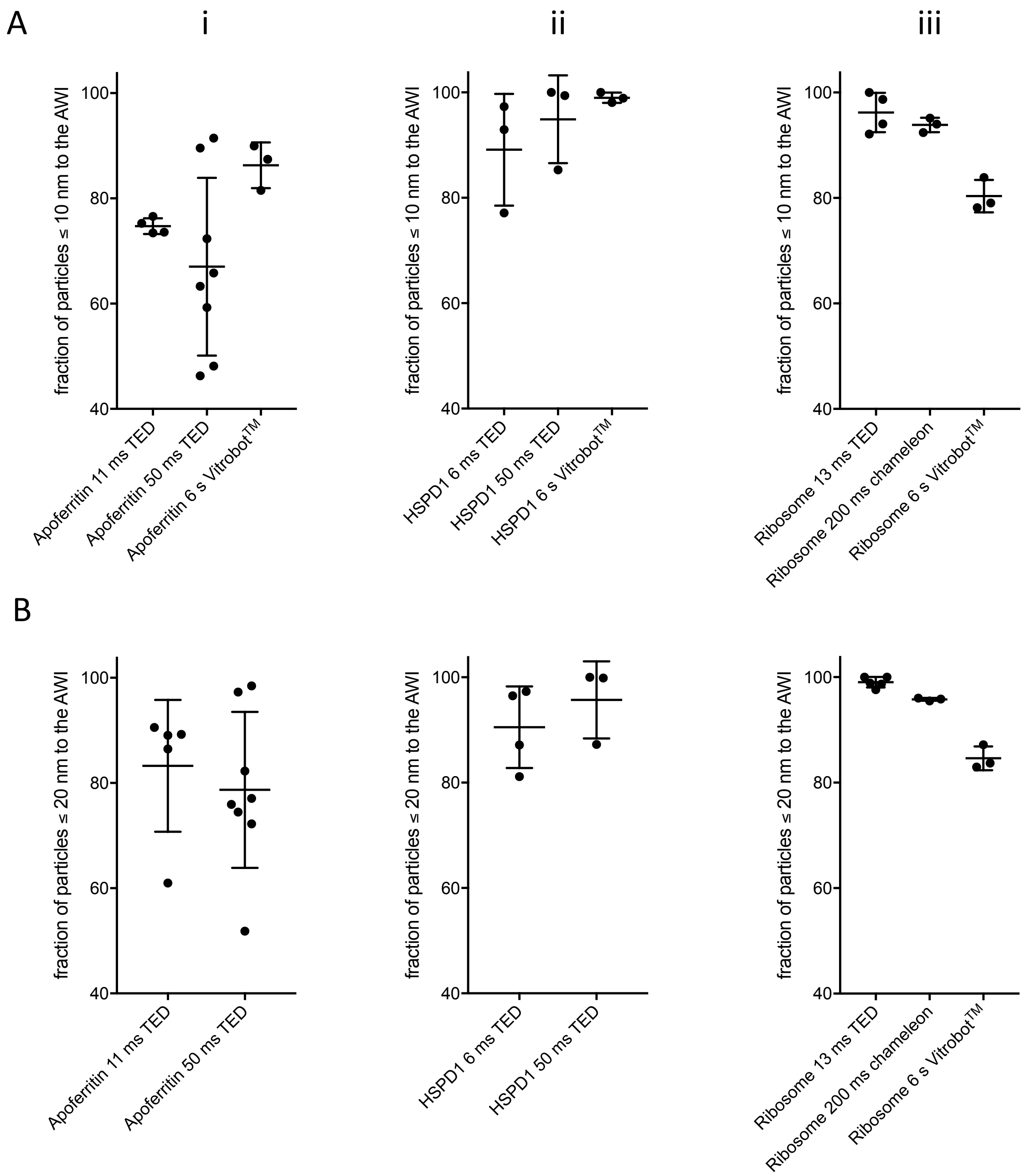

Figure S1, related to Figure 2. Partitioning of particles to the AWI. Fraction of particles partitioning to the AWI within 10 nm (A) or 20 nm (B) for apoferritin (i), HSPD1 (ii) and ribosomes (iii), with time and method of vitrification indicated. Some Vitrobot^{TM} values were excluded from (B) because of low ice thickness. Shown are the individual data points, mean value and standard deviation.

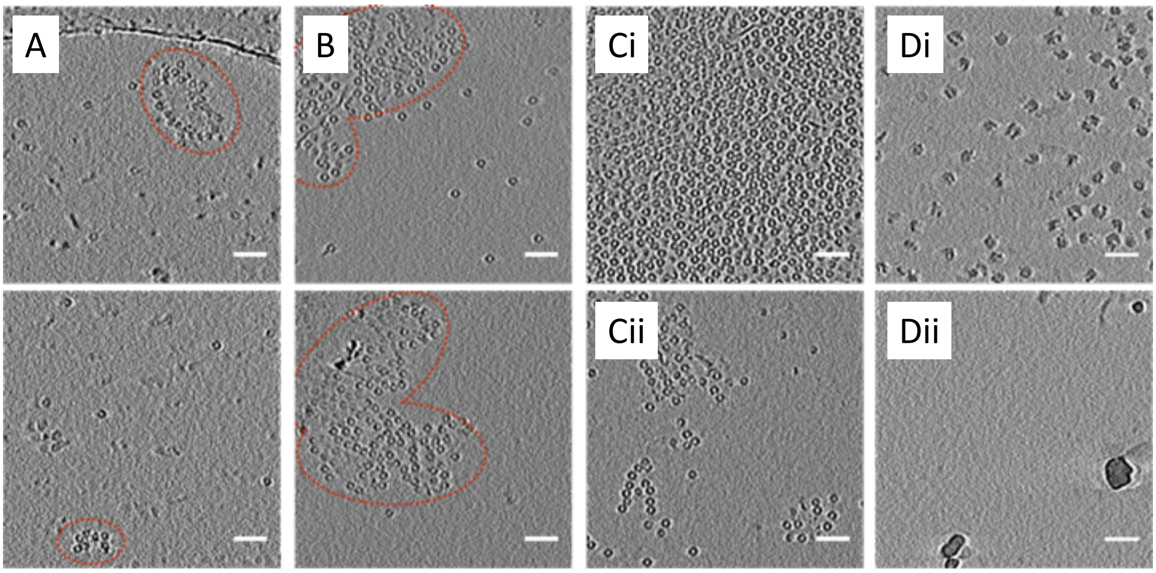

Figure S2, related to Figure 2. Surface aggregates at varying timepoints. Sections through reconstructed tomograms from TED grids, showing morphology of apoferritin aggregates at the AWI at (A) 11 ms or (B) 50 ms, outlined in red. Some tomograms showed highly asymmetric distributions of particles, with two interfaces from the same tomogram shown in (Ci) and (Cii) or (Di) and (Dii) for the apoferritin 50 ms and ribosome 13 ms sample, respectively. Scale bars 50 nm.

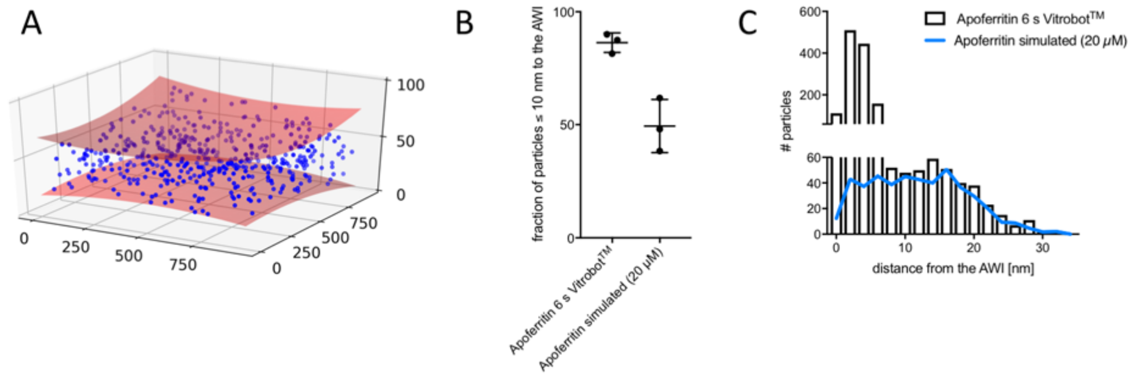

Figure S3, related to Figure 3. Comparing modelled data to experimental apoferritin partitioning. (A) Modelled distribution of particles (blue) in an ice layer (red), with coordinates indicated on axis (nm). AWIs were taken from an experimental tomogram and particle coordinates were randomly generated. This models a situation with no changes in concentration compared with sample applied, and no affinity for the AWI. (B) Comparison between modeled and experimental data for particle partitioning to the AWI. Particle concentration is 20 μM, P = 0.007. (C) Distributions of distances between particles and AWI for simulated (blue line) and experimental data (black bars) for apoferritin Vitrobot™ grids.

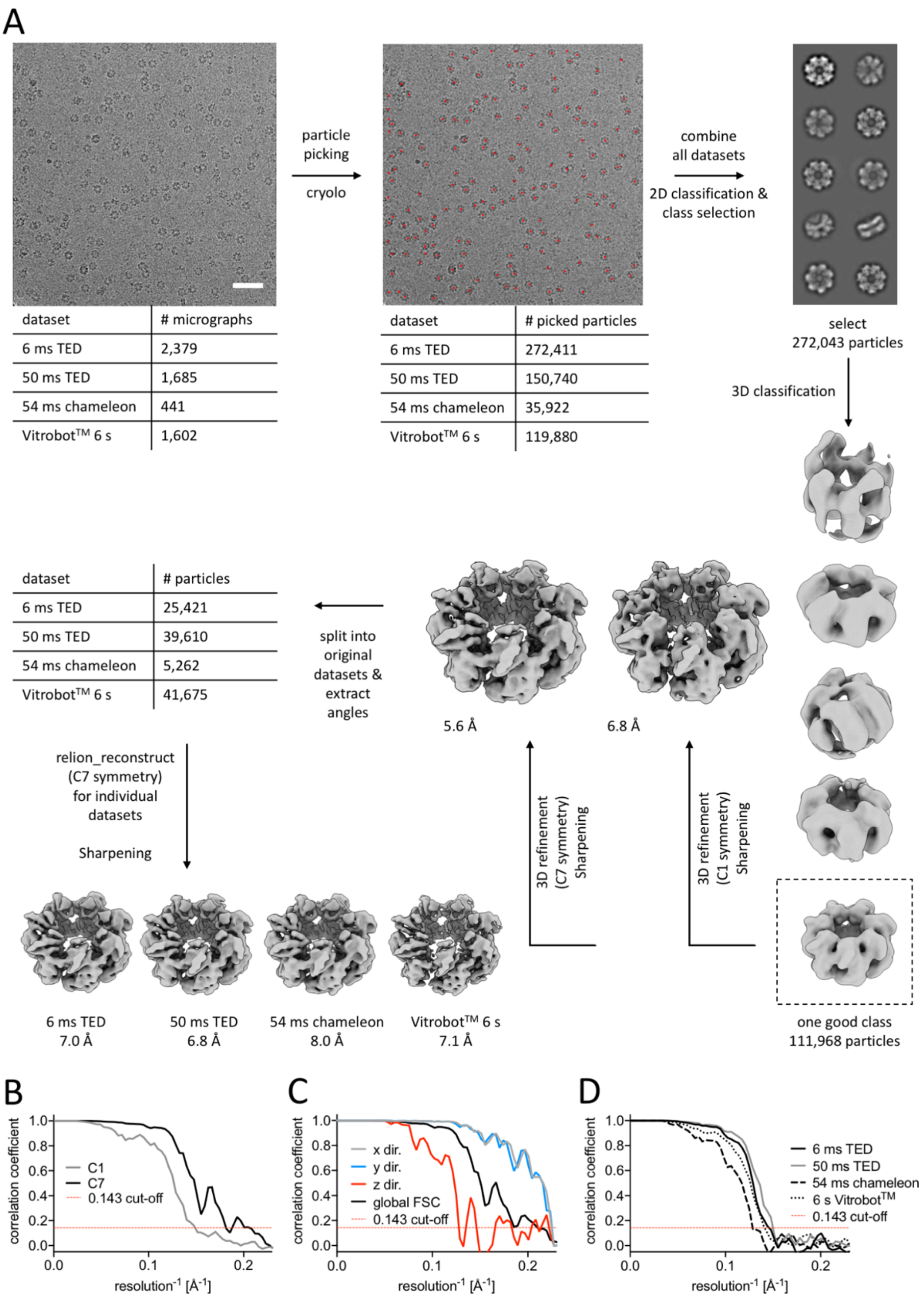

Figure S4, related to Figure 4. CryoEM image processing of HSPD1 data to yield angular distribution data and FSC curves for HSPD1 consensus structures. A Data processing pipelines for HSPD1 data showing particle numbers and corresponding 3D density maps and resolution for each sample preparation device and timescale analysed. Datasets have varying ice thicknesses, particle number and angular orientation and so resolutions cannot be directly compared. **(**B) FSC curves for masked maps of the consensus structure (all datasets combined) with and without symmetry. (C) 3D-FSC analysis of the same reconstruction, showing that resolution in the z-direction is limited through the lack of side views. Note that pixel size is 2.13 Å. (D) FSC curves for reconstructions for each individual dataset.

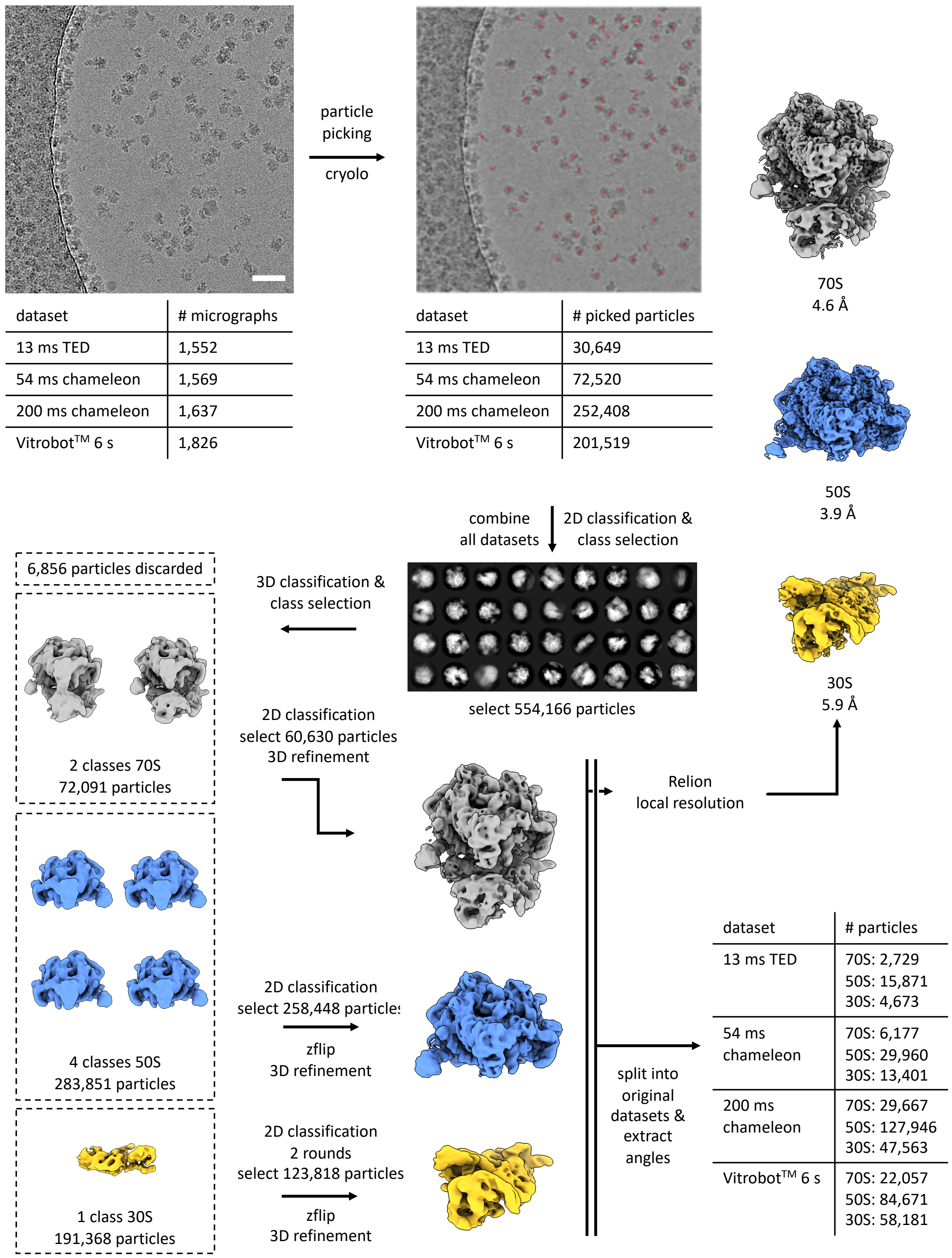

Figure S5, related to Figure 5. CryoEM image processing of ribosome data to yield angular distribution data. Data processing pipelines for ribosome angular orientation maps. Micrographs shown are representative of the type of micrograph used.

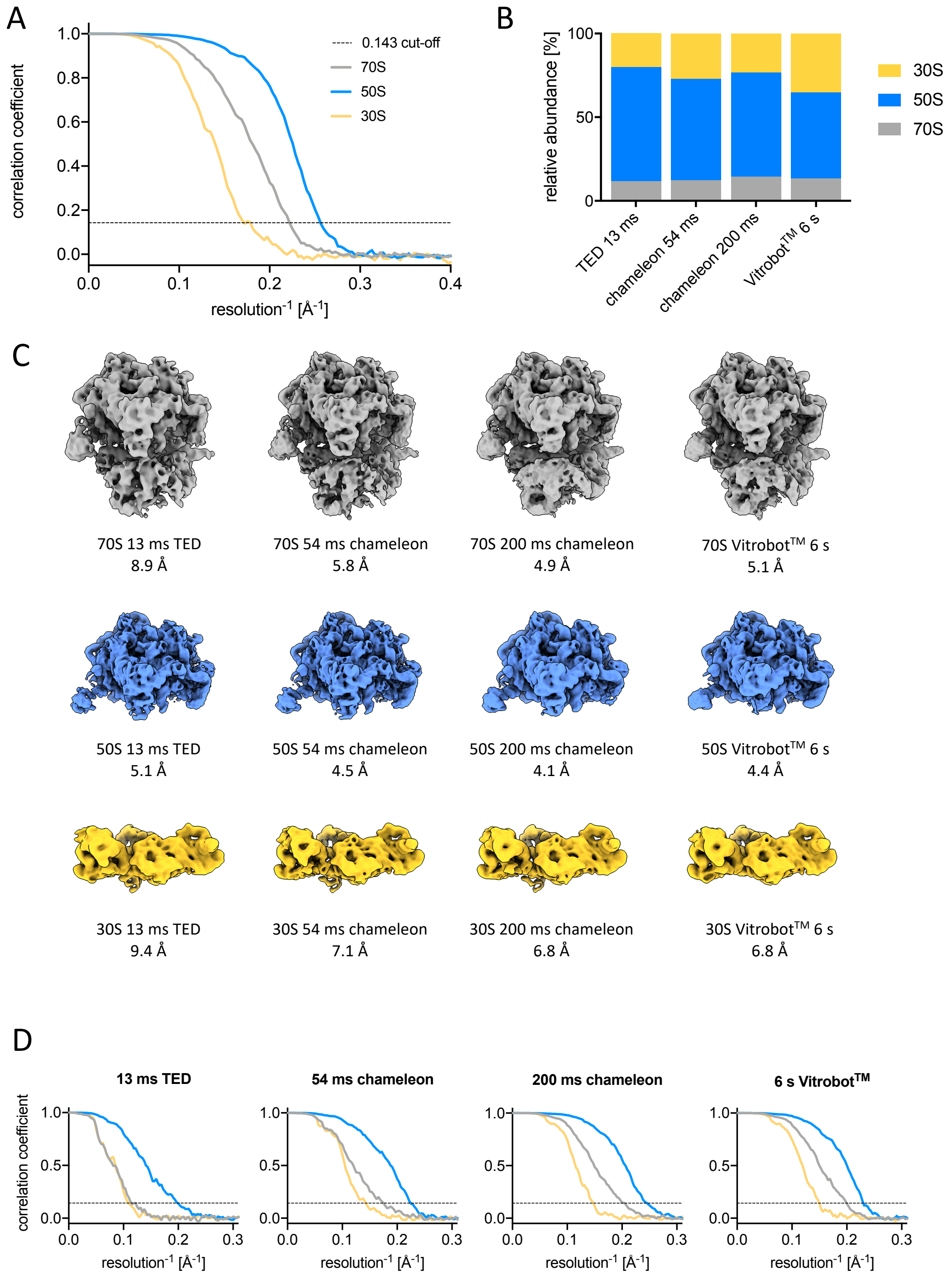

Figure S6, related to Figure 5. CryoEM reconstructions of ribosome data at different time points and vitrification devices. (A) FSC curves for masked, consensus reconstructions of 70S, 50S and 30S ribosome. (B) Relative particle numbers for 30S, 50S and 70S by individual dataset. (C) Individual reconstructed maps for 70S, 50S and 30S for all 4 subsets and corresponding FSC curves (D).

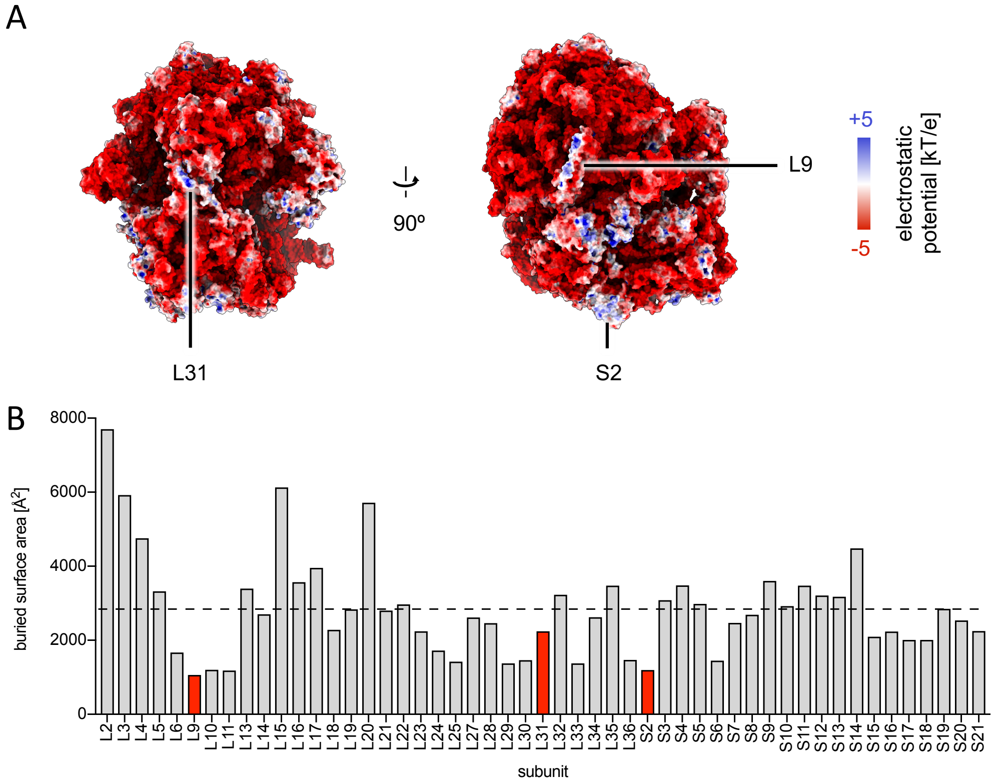

Figure S7, related to Figure 6. Analysis of the surface properties and buried surface area for the ribosomal proteins L9, L31 and S2. (A) Electrostatic surface potential of the ribosome with subunits L9, L31 and S2 highlighted showing their contrasting neutral/positive charge compared to the predominantly negative charge of the ribosome. (B) Buried surface are for the different ribosomal proteins with the average buried surface area indicated by a dashed line.

	Time and vitrification device	Repeat	Concentration [µM]	Applied concentration [µM]	Ice thickness [nm]	# particles	relative particle # (≤ 10nm)	relative particle # (≤ 20nm)
Apoferritin	11 ms TED	1	1.0	20.0	168	100	21*	61
		2	1.9		96	111	77	86
		3	1.4		78	64	73	89
		4	0.9		97	53	74	91
		5	2.2		139	186	75	89
	50 ms TED	1	3.4	20.0	152	253	72	77
		2	7.7		135	509	66	74
		3	2.1		68	54	48	72
		4	1.7		76	79	63	82
		5	1.4		64	54	59	76
		6	119.2		47	2160	90	98
		7	41.8		90	1835	91	97
		8	1.3		88	54	46	52
	6 s Vitrobot™	1	53.9	20.0	54	1751	90	97**
		2	69.1		43	1622	82	96**
		3	83.8		34	1582	87	99**
HSPD1	6 ms TED	1	1.5	11.0	95	70	77	87
		2	1.2		73	69	46*	81
		3	0.9		82	37	97	97
		4	2.3		62	85	93	96
	50 ms TED	1	18.8	11.0	60	680	99	100
		2	4.9		87	204	85	87
		3	7.4		73	325	100	100
	6 s Vitrobot™	1	18.9	0.6	39	193	100	100**
		2	10.0		50	270	99	100**
		3	9.3		64	156	98	100**
Ribosome	13 ms TED	1	1.3	2.5	113	57	19*	100
		2	0.3		130	14	100	100
		3	3.2		151	84	94	98
		4	3.9		119	178	92	99
		5	5.9		67	152	99	99
	200 ms chameleon	1	11.9	2.5	136	864	95	96
		2	12.5		137	934	94	96
		3	15.8		135	1235	92	96
	6 s Vitrobot™	1	17.0	0.8	92	601	79	84
		2	23.5		93	843	84	87
		3	20.2		97	787	78	83

Table S1, related to Figure 2. Summary table of tomograms analysed to produce partitioning and particle concentration data. "Concentration" is the estimated concentration from the tomogram, "Applied concentration" is the concentration of the sample applied. "Relative particle #" is the percentage of particles within ≤ 10 or 20 nm of the AWI.

* Values were excluded from analysis because of poor fitting of AWIs.

** Values were excluded from analysis because of low ice thickness.

Microscope	Titan Krios II
Magnification	42,000
Voltage (kV)	300
Electron dose per image ($e^-/Å^2$)	1.8 - 1.9
Fractions per image	3
maximum/minimum tilt (increment)	-60°, +60° (2°)
Tilt scheme	bidirectional
Defocus range (µm)	-5 to -10
Pixel size (Å)	3.4

Table S2, related to Figure 3. Microscope parameters for collection of cryo-ET data.

	**HSPD1**			
	**6 ms TED**	**50 ms TED**	**54 ms chameleon**	**6 s Vitrobot™**
Microscope	Titan Krios I			
Magnification	75,000			
Voltage (kV)	300			
Total electron dose ($e^-/Å^2$)	81	81	74	75
Exposure time	1.5	1.5	1.5	1.5
Number of frames	59	59	59	59
Defocus range (µm)	-2 to -4	-2 to -4.5	-1.5 to -3.5	-1.3 to -3.3
Pixel size (Å)	1.065			

Table S3, related to STAR Methods. Data collection parameters for SPA datasets of HSPD1.

	**ribosome**			
	**13 ms TED**	**54 ms chameleon**	**200 ms chameleon**	**6 s Vitrobot™**
Microscope	Titan Krios I			
Magnification	75,000			
Voltage (kV)	300			
Total electron dose ($e^-/Å^2$)	77	74	74	78
Exposure time	1.5	1.6	1.5	1.5
Number of frames	59	59	59	59
Defocus range (µm)	-1.3 to -3.3	-1.3 to -3.3	-1.3 to -3.3	-1.3 to -3.3
Pixel size (Å)	1.065			

Table S4, related to STAR Methods. Data collection parameters for SPA datasets of the ribosome.
